# Pathways to conspiracy: The social and linguistic precursors of involvement in Reddit’s conspiracy theory forum

**DOI:** 10.1371/journal.pone.0225098

**Published:** 2019-11-18

**Authors:** Colin Klein, Peter Clutton, Adam G. Dunn

**Affiliations:** 1 School of Philosophy, Australian National University, Canberra, Australia; 2 Centre for Health Informatics, Australian Institute of Health Innovation, Macquarie University, Sydney, Australia; Poznan University of Technology, POLAND

## Abstract

Many individuals who engage with conspiracy theories come to do so through a combination of individual and social factors. The interaction between these factors is challenging to study using traditional experimental designs. Reddit.com is a large connected set of online discussion forums, including one (r/conspiracy) devoted to wide-ranging discussion of conspiracy theories. The availability of large datasets of user comments from Reddit give a unique opportunity to observe human behavior in social spaces and at scale. Using a retrospective case control study design, we analyzed how Reddit users who would go on to engage with a conspiracy-related forum differed from other users in the language they use, differences in the social environments where they posted, and potential interactions between the two factors. Together, the analyses provide evidence for self-selection into communities with a shared set of interests which can feed into a conspiratorial world-view, and that these differences are detectable relative to controls even before users begin to post in r/conspiracy. We also suggest that survey-based and experimental studies may benefit from differentiating between passive private endorsement by individuals and active engagement with conspiracy theories in social spaces.

## Introduction

Conspiracy theories—beliefs attributing agency over important world events to the secret plotting of powerful, malevolent groups—have been common in our population over a sustained period [1–5]. Conspiracy beliefs have the potential to cause harm both to the individual and the community. Conspiracy endorsement is associated with lowered intention to participate in social and political causes [6], unwillingness to follow authoritative medical advice, increased willingness to seek alternative medicine [7–8], and a tendency to reject important scientific findings [9–10].

There are a variety of attitudes individuals might have towards conspiracy theories. Many people passively *endorse* conspiracy theories, in the sense that they will assent to one or more conspiracy-related beliefs if asked. Conspiracy endorsement can be a relatively weak attitude, reflecting a general suspicion of the powerful [11]. Measurement of assent also appears to be strongly influenced by contextual and partisan cues [12]. A subset of individuals who endorse conspiracy theories also actively *engage* with conspiracy theories by, for example, discussing and spreading them online.

Many researchers take people who endorse or engage with conspiracy theories to depart from the ordinary norms of belief formation. As such, there has been a search for psychological factors which explain why particular individuals find conspiracy theories attractive. The psychological literature offers two types of explanation. *Cognitive* explanations cite mental processes that are adaptive in some contexts (including evolutionary ones), but which go awry in contemporary political situations [13]. Such processes might include Bayesian [14] or abductive [15–16] inference about hidden causes, or over-enthusiastic pattern completion [17]. Such processes are in some sense universal, but exaggerated instances play an especially important role in explaining conspiracy endorsement in individuals. *Trait* explanations, by contrast, focus on factors which explain individual differences in endorsement of conspiracy theories. While both are important for understanding why people engage with conspiracy beliefs, trait explanations have received more attention: the wide variation in acceptance of conspiracy theories among individuals, combined with the negative consequences of that acceptance, is a natural explanatory target.

An early and influential set of trait theories focused on the attraction of conspiracy theories to the powerless. Hofstadter wrote of the attraction of conspiracy theories to those who “…see only the consequences of power—and this through distorting lenses—and have no chance to observe its actual machinery” [18, p. 86]. More recent literature has focused on a positive relationship with measures of powerlessness and external locus of control [19], the relationship between feelings of powerlessness and cognitive factors such as illusory pattern perception [20], and the role of stressful life events [21]. These accounts are not always critical of conspiracy endorsement: some assign it an important role in institutional critique by the politically disadvantaged [22] or emphasize the role that conspiracy theorizing may play in masking more salient social tensions [23].

A general role for distrust of and defiance towards authority has also been posited [24]. Goetzel [3] identified lack of interpersonal trust as a key predictor of conspiratorial belief. Goetzel also noted a close relationship between endorsing conspiracy theories and being a member of a racial minority. The relevant conspiracy theories often resemble legitimate reasons for distrust by a minority community—for example, the theory that human immunodeficiency virus (HIV) was engineered to decimate African-American communities appears to be more popular among those aware of the Tuskegee Syphilis experiments and other historical medical abuses of African-Americans [25–27]. In such theories, the driving role is often standing negative emotions directed towards the powerful: anger, disgust, or paranoia.

A handful of explanations consider conspiracy beliefs to be pathological, placing them on a spectrum that includes paranoid ideation, paranormal belief, and schizotypy [28–30].

A further cluster of theories focuses on the role of factors such as individual self-esteem in the face of difficult life circumstances [31,32] or in positive individuation from others [33], emphasizing the role that conspiracy endorsement can play in these processes. Conspiracy theories can provide exculpatory narratives for individual hardship. The narrative of how one came to believe in conspiracy theories can also be a powerful anchor for identity, functioning for the individual as a kind of “transformative experience” [34]. Belief in conspiracy theories appears to correlate with a need for uniqueness [35]. Drawing on qualitative work, Franks, Bangerter, Bauer, Hall, & Noort [36] suggest that conspiracy engagement may be part of an optimistic worldview that focuses on personal and social growth.

Recent literature also suggests that conspiracy endorsement may constitute a distinct construct which correlates with a variety of more traditional personality traits [37,38]. One popular way of cashing out this construct is in terms of a *conspiratorial worldview*, in which people who endorse one conspiracy theory are more likely to endorse others [39]. Goetzel [3] suggested that conspiracy endorsers tend towards a “monological belief system” in which beliefs in any two conspiracy theories tend to be incorporated under a common umbrella [39–42].

Trait theories do not need to be mutually exclusive; several may provide useful and co-existing explanations for why people endorse conspiracy theories [43,6,24].

## Social self-selection

Psychological theories of conspiracy endorsement tend to focus on the individual abstracted from their social context. While it is clear that social context plays a role in shaping conspiracy belief endorsement in individuals, studies examining social factors associated with conspiracy belief are comparatively rare [44]. Yet social effects undoubtedly exist. Social groups affect whether ambiguous information is interpreted in a conspiratorial manner [45–46]. Studies examining the structure of communication patterns within social networks have considered how *homophily* can affect the way beliefs spread and persist [47–48], how beliefs can be distorted through collective memory [49], and how it can exacerbate the spread of misinformation in particular [50].

These studies focus on the effect of network structure rather than individual differences in personality and psychology. This should not be construed as a process whereby individuals are passively embedded in a social space. Individuals who endorse conspiracy beliefs are known to seek out others with shared beliefs [51], which means that active social self-selection may provide a plausible mechanism for how people assimilate multiple conspiracy theories within a conspiratorial worldview.

The relationship between self-selection and stable traits taps into an old debate in both personality and social psychology. An individual’s behavior depends both on their intrinsic dispositions and on the situations in which they find themselves. At short timescales, the interaction between personality and situation is widely accepted [52]. Longer timescales present opportunities for more complex interactions. As Allport [53] noted, personality determines which situations people will embrace and which they will avoid. In Funder’s [54, p. 575] pithy formulation, “…while a certain kind of bar may tend to generate a situation that creates fights around closing time, only a certain kind of person will choose to go to that kind of bar in the first place.”

Buss [55] distinguished three processes at work in long-term interactions. Individuals *select* a social milieu, which in turn *evokes* certain responses from them given their traits, and over the long run they *manipulate* their social surroundings to create and reinforce a niche. Emmons, Diener, and Larson [56] similarly distinguished *choice* mechanisms and *affect* mechanisms. In the former, individuals’ personalities lead them to consciously seek or avoid certain kinds of situation, while in the latter, people merely prefer situations that fit with their personalities and so are reinforced for choosing appropriately.

While these longer-term interactions are important, studying them presents unique challenges [57]. Continuous recording of reactions is only possible over relatively short time periods in the lab. The study of longer-term interactions has primarily been approached by intermittent experience sampling [58,59] or varieties of retrospective self-report [60,61]. Both techniques provide valuable evidence but face well-known methodological challenges Individuals who actively engage with conspiracy theories in social spaces are also challenging to study using experimental designs. Conspiracy engagement often comes with skepticism about official motives, making it difficult to recruit participants. There is also a risk of selection bias in recruitment, as a small subset of conspiracy engagers tend to be disproportionately visible [62].

## Methodological innovation: Online datasets

The availability of large datasets from online social media offers a unique opportunity to observe longitudinal interactions between social groups and individual traits [63]. Participation in online forums is typically open and voluntary, allowing individuals considerable latitude in selecting their social environment.

In addition, online forums provide a much larger source of data for analysis, providing enough power to examine a larger number of factors at once. The sheer size of some corpora allows for effective unsupervised analyses [64], avoiding the coding issues present in traditional survey designs. While they are restricted to studying associations rather than experimentally manipulated effects, large observational datasets can be used to generate new hypotheses and guide future research designs.

Studies examining or simulating the behaviors of people expressing conspiracy beliefs online have primarily been focused on how the spread of conspiracy beliefs are facilitated by network structure [65–67]. Social reinforcement and homophily play an important role in this spread, a fact that has been demonstrated both by modeling [68] and observational studies [69,70] of social networks. Community feedback and reinforcement also plays an important role in shaping users’ actions in online forums [71,72].

One important source of online conspiracy theorizing is the website Reddit.com (or ‘Reddit’). Reddit is a network of around 1.2 million online forums (known as *subreddits*), with around 330 million monthly active users. Reddit data have been used to examine the structure of conversations and propagation of information [73], and hateful and offensive speech [74–76], in addition to general linguistic analyses [77,78].

Reddit has also been identified as a key part of the “propaganda pipeline” [79] that amplifies conspiracy theories on their way to more visible websites (such as Facebook) and mainstream media [80]. Reddit includes a dedicated subreddit (r/conspiracy) for discussing conspiracy theories. Examination of the comments that Reddit users who post in r/conspiracy therefore provides a unique window into a socially significant subset of individuals who actively engage with conspiracy theories in a social space.

## The present research

There is at least one study about how Reddit users interact within the r/conspiracy subreddit after salient events [81], and one which examines the diversity of interests among Reddit users who posted to an online forum for conspiracy beliefs [62]. However, we know of no studies examining their behavior over time and before they first post in r/conspiracy. Our aim was to examine what makes Reddit users who would go on to engage with conspiracy theories different from other Reddit users.

We undertook an exploratory analysis using a case control study design, examining the language use and posting patterns of Reddit users who would go on to post in r/conspiracy. (the *r/conspiracy group*). We analyzed where and what they posted in the period preceding their first post in r/conspiracy to understand how personal traits and social environment combine as potential risk factors for engaging with conspiracy beliefs.

Our goal was to identify distinctive traits of the r/conspiracy group, and the social pathways through which they travel to get there. We compared the r/conspiracy group to matched controls who began by posting in the same subreddits at the same time, but who never posted in the r/conspiracy subreddit. We conducted three analyses. First we examined whether r/conspiracy users were different from other users in terms of *what they said*. Our hypothesis was that users eventually posting in r/conspiracy would exhibit differences in language use compared to those who do not post in r/conspiracy, suggesting differences in traits important for individual variation. Second, we examined whether the same set differed from other users in terms of *where they posted*. We hypothesized that engagement with certain subreddits is associated with a higher risk of eventually posting in r/conspiracy, suggesting that social environments play a role in the risk of engagement with conspiracy beliefs. Third, we examined language differences after accounting for the social norms of where they posted. We hypothesized that some differences in language use would remain after accounting for language use differences across groups of similar subreddits, suggesting that some differences are not only a reflection of the social environment but represent intrinsic differences in those users.

## Materials and methods

### Participants

#### Users and ethics approval

Our study participants were Reddit users who posted comments to online forums between 2007 and 2015. They were selected from a publicly available dataset comprising 1.10 billion comments from 1,419,406 users posted to 224,625 subreddits between October 2007 and May 2015 (see S1 Appendix).

Participants whose data was used were not contacted. The data were originally collected and made available under the terms permitted by the Reddit Terms of Service. As we were using publicly available data examined and reported in aggregate, ethics exemptions were granted by both Macquarie University and the Australian National University.

#### Selection of users

Reddit allows posts by automated programs known as *bots*, which post comments in ways that can skew descriptive statistics. To remove accounts associated with bots, we first looked at each poster in a target set of subreddits (including r/conspiracy) and calculated the number of other subreddits in which they posted (their forum diversity). A list was compiled of usernames whose forum diversity was more than 15 standard deviations above the mean. Manual inspection revealed that every member of this list was probably a bot, whereas more aggressive cuts also included posters who were clearly human. This was combined with a list of usernames corresponding to known bots posted on Reddit itself (See S1 Appendix). This process identified 466 bots, which were excluded from subsequent analyses.

The r/conspiracy group was defined as the set of users posting at least 3 comments in r/conspiracy, and at least 4 times in each of the six contiguous 30-day periods immediately prior to their first post in r/conspiracy. Users who posted in r/conspiracy but did not meet both criteria were excluded from the analysis. All comments posted by included users made before their first post to r/conspiracy were included in the subsequent analysis, and these were used to characterize their language use and topics of interest. Posts by this group after their first post to r/conspiracy were not used in the analysis.

#### Manual validation

Reddit has its own culture with a complex set of norms. Some users post in r/conspiracy because they want to debunk conspiracy theories, while others enjoy “trolling” by deliberately provoking conspiracy theorists. Previous work on r/conspiracy suggested that between 4% and 12% of posters in r/conspiracy might fall into one of these categories [62]. We manually examined the posts of 100 identified r/conspiracy users and determined that at most 9 of them were consistently either skeptical or non-serious. We took this to be an acceptable noise rate. Posters in r/conspiracy who do not engage seriously with conspiracy theories should be expected to be more like other posters on Reddit; at most, then, the presence of such individuals in our dataset would only reduce sensitivity, rather than create false positives.

#### Matched controls

Reddit is a diverse community. Many differences between the average Reddit user and a user posting in r/conspiracy may simply reflect that diversity. To minimize spurious differences, we constructed a control group by matching target users to posters whose first post was in the same forum at nearly the same time. Each user’s matched control thus “enters” our dataset at the same place and time but ends up on a different trajectory. To construct the *matched control group*, we first created a candidate control group by identifying users who never posted in r/conspiracy, and who had posted at least 4 times in any 6 contiguous 30-day periods. From the candidate control group, we then constructed matched pairs for each user in the target group. For each r/conspiracy poster, we identified their first post on Reddit. We then identified users from the candidate group whose first post was in the same subreddit within 24 hours of the r/conspiracy poster. We then iteratively assigned the user whose first post was closest in time to the first post of the r/conspiracy poster, under the constraint that matches had to be unique. We examined only comments between their first post and the final post of their matched control. To ensure matched controls had enough posts to reliably compare, we eliminated 480 matched pairs in which the control user had not posted at least 35 comments in that restricted timespan. This process identified 15,370 users in each group (30,740 total), which were used for all subsequent analyses.

#### Preprocessing

Reddit posts consist of an initial post followed by nested comments underneath. The dataset includes only the nested sets of comments that follow the original (“link”) posts, not the link posts themselves. These comments and their associated metadata were the basis for analysis.

When analyzing the r/conspiracy users, we examined only comments from their first post to their final post before posting in r/conspiracy. When analyzing matched controls, only comments posted in the period between their first post and their matched partner’s first post in r/conspiracy were considered.

We pre-processed comments posted by included users to remove escape characters, URLs, and any lines which began with a ‘>‘ (which is typically used to mark text quoted from another author). Comments with fewer than 3 words after processing were omitted. We then concatenated each user’s comments, with subsequent analyses performed on a per-user basis.

### Measures

#### Language use

Psychological traits shape the language that individuals use. Computational analysis of language usage has been successfully used to investigate personality traits [82,83] as well as individual differences in emotionality and social relationships [84]. Linguistic features are a good marker of whether a discussion will be constructive, both experimentally [85] and on Reddit [86]. Computational analysis of word use within the r/conspiracy forum has provided evidence about common narrative structures of conspiracy engagement [87] and individual differences in user’s interests [62].

To measure word use across particular language categories we used Empath [88,89], an open-source Python package which extracts linguistic characteristics from written text. Empath categories are built in a multi-stage process. First, categories and corresponding seed words are derived from pre-existing semantic knowledge bases such as ConceptNet. A vector space model is then trained on a large corpus of text, including Reddit comment data, and each category is expanded to include terms which occur near seed terms in the vector space model, indicating semantic similarity. Finally, categories are pruned via human inspection to eliminate intruders [82,83]. Although we used pre-defined Empath categories, the library allows for expansion to other user-defined categories via the same procedure, making it a flexible tool for examining textual data.

Empath scores themselves are weighted word frequencies across members of the category. Empath normalizes for aggregate comment length, returning frequency counts per category as the primary data. Empath scores are highly correlated (r = 0.91, see [88]) with those of the more widely-known LIWC Linguistic Inquiry and Word Count (LIWC) package where they overlap. Empath has a broader range of empirically derived lexical categories than LIWC. Further, its categorization scheme was partly trained on Reddit data, so it has found significant use in linguistic analysis of online discussion, including the spread of hate speech on Twitter [90–92] and YouTube [93], and the interface between media and technology [94,95]. Empath has also been used specifically to study Reddit, including community growth [96] and self-expressions of mental illness [97].

Empath evaluates a large number (194) of lexical categories, many of which are irrelevant to the present study. We focused on a subset of 85 categories corresponding to 6 different psychological theories about the antecedents of conspiracy belief (Table 1). To determine which lexical categories were included for each factor, the three authors each independently chose candidates; categories chosen by at least 2 of the three raters were included. Each of the proposed theories could themselves be operationalized in a variety of ways, and so the procedure was designed to err on the side of inclusion.

**Table 1:** Lexical categories used from Empath mapped to factors associated with conspiratorial belief.

Factor	References	Lexical Categories
Powerlessness/Outlet for negative feelings	[18,19,20,22]	*aggression*, *anger*, *anonymity*, *confusion*, *deception*, *dominant_heirarchical*, *fear*, *government*, *hate*, *help*, *independence*, *leader*, *negative_emotion*, *nervousness*, *pain*, *poor*, *positive_emotion*, *power*, *pride*, *rage*, *strength*, *suffering*, *swearing_terms*, *timidity*, *torment*, *trust*, *weakness*
Defiance and Distrust	[24]	*aggression*, *anger*, *anonymity*, *communication*, *deception*, *disappointment*, *disgust*, *dispute*, *dominant_heirarchical*, *emotional*, *exasperation*, *fear*, *hate*, *help*, *independence*, *leader*, *negative_emotion*, *rage*, *ridicule*, *suffering*, *swearing_terms*, *trust*, *violence*
Maintenance of self- esteem	[31,32]	*achievement*, *affection*, *aggression*, *anger*, *anticipation*, *disappointment*, *dominant_heirarchical*, *emotional*, *envy*, *fun*, *healing*, *independence*, *joy*, *leader*, *love*, *negative_emotion*, *neglect*, *optimism*, *poor*, *positive_emotion*, *power*, *sadness*, *strength*, *sympathy*, *trust*, *valuable*, *warmth*, *weakness*
Personal values and individuation	[33]	*blue_collar_job*, *dominant_personality*, *economics*, *family*, *friends*, *healing*, *health*, *heroic*, *home*, *independence*, *occupation*, *order*, *philosophy*, *politics*, *positive_emotion*, *power*, *pride*, *religion*, *trust*, *valuable*, *white_collar_job*, *worship*
Psychopathology	[28,29]	*affection*, *aggression*, *confusion*, *death*, *emotional*, *fear*, *healing*, *health*, *help*, *medical_emergency*, *negative_emotion*, *nervousness*, *pain*, *prison*, *rage*, *sexual*, *shame*, *suffering*
Conspiratorial world-view	[3,39–41]	*banking*, *business*, *communication*, *crime*, *deception*, *dominant_heirarchical*, *economics*, *government*, *internet*, *journalism*, *law*, *military*, *money*, *order*, *politics*, *power*, *prison*, *real_estate*, *religion*, *royalty*, *science*, *social_media*, *stealing*, *terrorism*, *trust*, *violence*, *war*, *wealthy*, *white_collar_job*

For each category, we examined whether there was a significant difference in posting frequency of terms in that category between the r/conspiracy group and the group of matched controls, using Welch’s t-test with an alpha of 0.01 (corrected for multiple comparisons). To estimate the magnitude of the difference in effect, we calculated Cohen’s |*d|* (hereafter ‘*d*’). For this and subsequent analyses, we considered only terms which showed a significant difference and had *d*>0.2. These high-*d* factors were the bases for subsequent analysis.

#### Social environment

To determine which subreddits might represent important pathways through which users travel to reach r/conspiracy, we looked for over-representation of r/conspiracy users in the subreddits relative to the control group. To do this we examined each subreddit and counted the number of r/conspiracy users and matched control group users that had posted at least one comment. To avoid spurious results and potential re-identification of individual users, we analyzed the set of subreddits in which r/conspiracy and control group users had posted at least once during matched timespans, and a minimum of 100 users across both groups had posted at least once.

To be able to examine how language use differed within certain communities on Reddit, we grouped similar subreddits by *theme community*. We constructed a *similarity network* based on simple co-posting behaviors, without considering the chronology of the posts. Each subreddit was represented by a node in the network, with undirected edges between the nodes defined by the number of shared users (users who posted in both subreddits at least once) divided by the total number of users who posted in either subreddit (i.e. the Jaccard similarity [98]).

We then applied a community detection algorithm to the similarity network to group subreddits by theme based on the number of users they shared. Community detection algorithms are used to identify clusters of well-connected nodes in a network. Most algorithms aim to identify clusters by maximizing the number of connections within each community compared to the number of connections between communities. We applied the greedy modularity optimization method [99], which is commonly used for large networks. In this application, the algorithm starts with all subreddits in separate communities and then merges according to a gain in modularity—a measure of the density of connections within versus between communities. The number of communities is not specified in advance; rather, the algorithm stops when no further merging of communities improves modularity.

For each group of subreddits within a theme community we calculated two measures characterizing the differences between r/conspiracy users and the matched control group users. The user-count ratio was defined as the number of r/conspiracy users posting at least once in the constituent subreddits relative to the matched controls. The post-count ratio was defined by the total number of posts from r/conspiracy users in the constituent subreddits of the theme community compared to the number of posts from the matched control group users. Each value can be interpreted as a signal of the risk associated with posting to that subreddit.

#### Interactions between language use and social environment

For each high-*d* Empath factor identified in the language use experiment, and each theme community identified in the social environment experiment, we examined the contrast between language use by control and r/conspiracy users but restricted just to posts made in any of the subreddits in that theme community. As before, differences were tested by Welch’s t-test with an alpha of 0.01 (corrected), and Cohen’s *d* was used as an estimate of the magnitude of effect, where we again used 0.2 as a threshold for indicating a substantial difference.

## Results

The 15,370 r/conspiracy users and the 15,370 matched controls posted to a set of 38,797 unique subreddits, and of these 1,834 met our inclusion criteria for analysis. Within this set of subreddits, r/conspiracy users posted a median of 743 (interquartile range 333 to 1,749) comments, with a median of 20,599 (IQR 8,302 to 52,321) total postprocessed words of comments. Their matched controls posted a median of 300 (IQR 142 to 707) comments, and a median of 8,082 (IQR 3,407 to 21,068) total postprocessed words of comments. The results indicate that r/conspiracy users posted at a substantially higher rate. We were unable to measure whether the groups were posting at different times of day and we did not measure whether comments were spread across a broad set of posts or concentrated within longer conversations on individual posts.

### Language use

**Fig 1:**
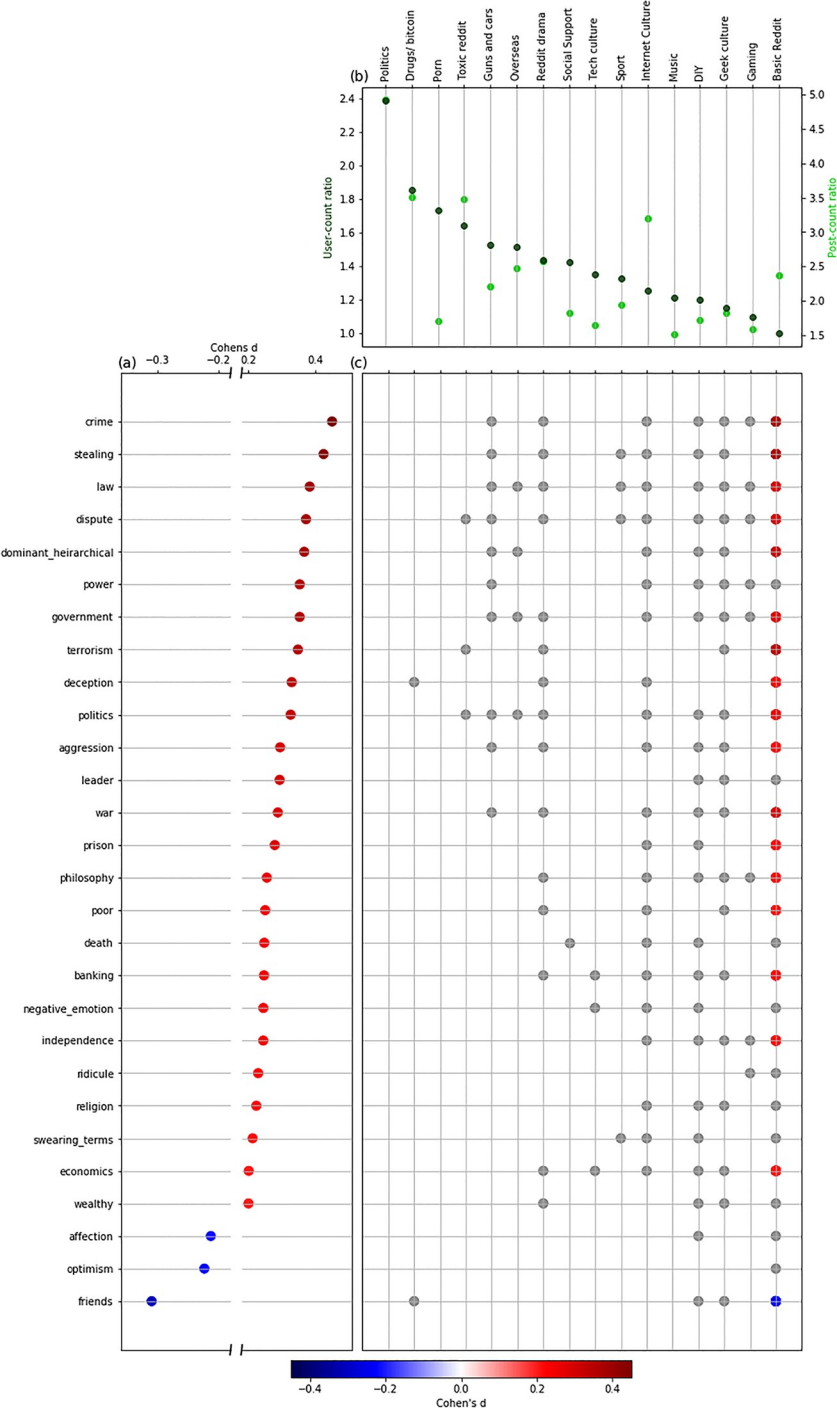
Differences between the r/conspiracy group and the matched controls. Differences between the r/conspiracy group and the matched control group by: (a) language use, including all Empath categories with significant positive (red) and negative (blue) differences and Cohen’s *d*>0.2; (b) posting differences by theme community given by user-count ratio and post-count ratio; and (c) differences in language use accounting for theme community. Positive (red) and negative (blue) differences are colored where Cohen’s *d*>0.2. Grey circles indicate significance which did not reach the effect size threshold.

From the set of 91 Empath categories included in the analysis, 75 exhibited a significant difference between r/conspiracy users and their matched controls. Among those with significant differences, 26 differences had *d*>0.2 (Fig 1a).

Where r/conspiracy group users posted certain terms more frequently than the matched control group users, the most prominent differences were in the Empath categories ‘crime’ (*d* = 0.45), ‘stealing’ (*d* = 0.43), and ‘law’ (*d* = 0.38). The categories of ‘dispute’, ‘dominant_hierarchical’, ‘power’, ‘government’, and ‘terrorism’ each produced *d*>0.35.

There were relatively few significant negative differences: the only categories where matched control group users posted certain terms substantially more frequently were in the Empath categories ‘friends’ (*d* = -0.31), ‘optimism’ (*d* = -0.22) and ‘affection’ (*d* = -0.21). These terms were drawn from the “Maintenance of self-esteem” and “Personal values and individuation” categories (Table 1), though the higher frequencies among the matched control group users is suggestive of alienation rather than positive bonding in r/conspiracy group users.

Other categories suggested by theories in Table 1 did not make the threshold for inclusion, typically because their effect size was too low. Notably absent are Empath categories encompassing specific negative affects like ‘fear’ (n.s.), ‘sadness’ *(d* = 0.09), and ‘nervousness’ (*d* = -0.1), for which the differences are either small or in the wrong direction from what would be expected given the theory.

### Social environment

When applied to the similarity network of 1,834 subreddits, the community detection algorithm identified 16 theme communities. The communities included between 44 (the ‘Basic Reddit’ theme community) and 334 subreddits (the “Gaming & Television” theme community). The communities were characterized and named by examining their top 10 subreddits and choosing names that indicated the typical contents of those subreddits (Table 2), revealing differences in the topics of interest. Some of the theme communities covered more than one area of interest (for example, the “Guns & Cars” theme community), while others were relatively closely related, which is apparent in a visualization of the complete network (S1 Fig).

**Table 2:** Theme communities.

Theme Community	Top subreddits
Guns & Cars	cars, motorcycles, Autos, CityPorn, TopGear, Justrolledintotheshop, MilitaryPorn, carporn, knives, aviation
Sport	nfl, sports, soccer, nba, hiphopheads, hockey, baseball, CFB, MMA, fantasyfootball
Toxic Reddit	Showerthoughts, mildlyinfuriating, pcmasterrace, TumblrInAction, Unexpected, thatHappened, CrazyIdeas, FanTheories, TrollXChromosomes, InternetIsBeautiful
Tech Culture	talesfromtechsupport, web_design, learnprogramming, techsupportgore, sysadmin, investing, google, SOPA, Ubuntu, Entrepreneur
Reddit Drama	AskHistorians, ShitRedditSays, Foodforthought, conspiratard, TheoryOfReddit, DepthHub, Enhancement, circlebroke, Feminism, fifthworldproblems
Internet Culture	gifs, LifeProTips, mildlyinteresting, 4chan, cringepics, woahdude, JusticePorn, ImGoingToHellForThis, reactiongifs, cringe
Overseas	unitedkingdom, dayz, australia, civ, europe, KerbalSpaceProgram, Eve, britishproblems, Planetside, polandball
Politics	Conservative, PoliticalDiscussion, ronpaul, TrueAtheism, DebateReligion, islam, progressive, PoliticalHumor, Republican, POLITIC
Geek Culture	offbeat, DoesAnybodyElse, programming, comics, self, geek, entertainment, scifi, apple, business
Pornography	RealGirls, NSFW_GIF, Boobies, Celebs, ass, PrettyGirls, japan, girlsinyogapants, nsfw_gifs, milf
Drugs & Bitcoin	Bitcoin, LucidDreaming, Psychonaut, treecomics, Paranormal, UFOs, dogecoin, Glitch_in_the_Matrix, horror, eldertrees
Music	listentothis, WeAreTheMusicMakers, Guitar, dubstep, Metal, electronicmusic, vinyl, ifyoulikeblank, classicalmusic, punk
Gaming & Television	Games, skyrim, buildapc, gameofthrones, pokemon, breakingbad, leagueoflegends, starcraft, techsupport, tf2
DIY	food, Frugal, TwoXChromosomes, TrueReddit, malefashionadvice, DIY, firstworldproblems, YouShouldKnow, loseit, Cooking
Basic Reddit	AskReddit, pics, funny, WTF, gaming, IAmA, todayilearned, videos, politics, worldnews
Social Support	relationships, relationship_advice, amiugly, AskMen, seduction, AskWomen, depression, confession, SuicideWatch, OkCupid

The sixteen theme communities with names and top 10 subreddits, as determined by total number of users from either group who posted at least once in the subreddit during the relevant time span.

The user-count ratios varied by theme community (Fig 1b). The highest user-count ratio was in the “Politics” theme community, where there were 2.4 times as many r/conspiracy users as control users that posted in at least one subreddit in the group. The lowest user-count risk ratio was for the “Basic Reddit” theme community, which included several of the most popular subreddits including “r/AskReddit”, “r/pics”, and “r/funny”, where nearly equal numbers of users from each group had posted at least once. For some theme communities, r/conspiracy users were consistently over-represented across most of the constituent subreddits, whereas other theme communities included smaller clusters of over-representation of r/conspiracy users among a larger set where there was less over-representation (S1 Fig).

The r/conspiracy group users tended to post more frequently across all theme communities (Fig 1b) but there were notable differences relative to the user-count ratios. For example, r/conspiracy users were over-represented in the “Pornography”, “Tech Culture”, and “Music” theme communities (posting across many of the subreddits at least once) but had relatively low post-count ratios suggesting they may have been less engaged with those communities. Conversely, in the “Internet Culture” and “Toxic Reddit” theme communities, r/conspiracy users were not only over-represented in the constituent subreddits but also had relatively high post-count ratios, suggesting ongoing and stronger engagement with the themes and with other users in the subreddits in those theme communities.

### Interactions

In the “Basic Reddit” theme community there were clear differences between the language use of the r/conspiracy group relative to the matched controls, and for 16 of the 26 Empath categories the difference was substantial (*d*>0.2). For example, users who would go on to post in r/conspiracy were much more likely to use “aggression” terms in the constituent subreddits in the “Basic Reddit” theme community.

Examining differences in language use within each of the theme communities indicates that there were significant differences between r/conspiracy users and matched control group users within most of the theme communities (Fig 1c), but these differences were not as clear as the overall differences in language use. The results suggest that many of the clear differences observed between the two groups in the overall language use analysis were likely to have been because of *where* the r/conspiracy users were posting rather than *what* they were posting.

For example, in the “Politics” and “Pornography” theme communities, where r/conspiracy users were heavily over-represented relative to their matched controls, there were no significant differences in any of the 26 Empath categories. The results were similar in the “Tech Culture” and “Social Support” theme communities, where r/conspiracy users tended to closely match the control group in language use.

## Discussion

### Language and social factors

The r/conspiracy group users exhibited clear differences from other similar Reddit users in terms of both where they posted and what they posted. These differences in language use and social environment provide support for some of the theories of conspiracy belief.

First, there were clear differences in overall language use between r/conspiracy group and the matched control group. Most of the Empath factors exhibiting strong differences were associated with prior literature suggesting that a “conspiratorial mindset” leads to endorsement of conspiracy theories (Table 1). In general, Empath factors for which we observed a clear positive difference were aligned with issues of hierarchy and abuses of power. Also notable were Empath categories like “deception” and “terrorism”, which can be linked to an idea that is central to many conspiracy theories: that of hidden enemies among us.

Some have argued that the key psychological feature of conspiracy theorists is a “monological belief system” in which everything connects to everything else [3,39,42]. Recent work on r/conspiracy suggests that users with monological belief systems are responsible for the majority of posts but make up only a small percentage of users [62]. However, these results are not necessarily incompatible. A monological belief system may simply be the most extreme, and most salient, version of a more general conspiratorial mindset. Further, one lesson from these results might be the need to distinguish factors which lead people to engage with conspiracy theories in the first place from the factors which distinguish more and less extreme engagement with conspiracy theories. This would fit well with recent work emphasizing the multidimensionality of conspiracy constructs [100].

We did not find evidence to support previous literature observing differences in personality traits or varieties of compensation or psychopathology. Where previous literature focused on negative emotional states as drivers of conspiracy theory endorsement, we only found evidence for the non-specific ‘negative_emotion’ Empath category (*d* = 0.24). Equally striking was the lack of difference in use of language related to anger, disaffection, or other compensatory emotions. This contradicts some of the accounts that focus on the hostility of conspiracy endorsers [101], but concords with more recent work that highlights the lack of hostility in comments from conspiracy endorsers. For example, Wood and Douglas [102] carried out a study of conspiracy related comments on a news website. Comments were divided into "conspiricist" (those arguing for a conspiratorial explanation of events) and "conventionalist" (those arguing for a conventional account of events) comments, with a focus on comments judged to be aimed at persuading others. These comments were then rated for tone. Interestingly, comments from conspiricists were rated as less hostile than the comments from "conventionalists". Our findings lend support to this conclusion. We found evidence that r/conspiracy users were less likely than the control group to use terms from Empath categories “affection”, “optimism”, and “friends”, which might be suggestive of alienation or social isolation [18,22].

Some of the divergence from previous findings may come from the use of matched controls. Our study compared Reddit users who would go on to post in r/conspiracy with users who began posting on Reddit at the same time and in the same subreddits. People who endorse conspiracy theories may appear angrier or more disaffected compared to a general population, but this may be more common across online discourse and Reddit users in general.

Importantly, Wood and Douglas [102] point out the need to distinguish the target and type of hostility: to whom and regarding what features is a comment hostile? Conspiracy theorists might often be hostile towards others for being "dupes" of the system; non-conspiracy believers might be hostile towards the perceived paranoia of conspiricists, or their propensity to creative, ad-hoc additions in order to shore up their theories, and so on. This is a potential confound in our study. Whereas Wood and Douglas first selected comments as either conspiricist or conventionalist, our study of conspiracy posters includes those who go on to argue against conspiracy theorists as well as for them. If non-conspiricists tend to be angrier towards those who forward conspiracy theories, this may affect what we found in the tone of users who ended up in the conspiracy forum versus those who did not. Moreover, as we have suggested, there may be a greater effect of anger in general on reddit, which could make communities look more similar on this variable. That said, we looked at hostile language across a variety of subreddits, not just conspiracy-focused ones, suggesting that hostility is not being driven solely by conspiracy-related factors.

There may also be important differences between the phenomena we have focused on and those that have been the focus of previous studies. As we discussed, we examined people who have sought out a forum dedicated to conspiracy theories who actively discuss and share thoughts on the topic. This might be a different phenomena to simply passively endorsing conspiracy theories when questioned about them. This might be relevant to our findings on powerlessness. One possibility is that the type of sharing and active engagement seen in the forum is itself a type of reclaiming of power, a place to put forward ones thoughts, help out one’s peers and the wider community to see the truth, and so on. Passive engagement, by contrast, may stem from or promote powerlessness (and would be difficult for this method to detect). This might potentially be a source of difference when it comes to results regarding feelings of powerlessness.

There was also a clear difference in the risk profiles of different theme communities. The highest risk by far was in the “Politics” theme community, where there were 2.4 times as many r/conspiracy users posting in the subreddits compared to the control group, and they posted 5 times as many comments overall. Though there was the appearance of a skew to the political right in the subreddits included in the “Politics” theme community, this group also includes subreddits such as r/progressive; as well as relatively neutral subreddits such as r/PoliticalDiscussion and debate-oriented subreddits like r/DebateReligion, which cater to a wide variety of political leanings. Some of the spread is likely due to the vigorous debate across political positions that characterizes Reddit, but it appears that political debate (broadly construed) is especially attractive to users who would go on to post in r/conspiracy.

A useful framework that encompasses both the language use and social environments was suggested by Douglas and Wood [103,102,40], who note that endorsement of certain first-order conspiracy beliefs seems to be mediated by higher-order beliefs about the existence of cover-ups. Similarly, McCauley and Jacques [14] suggest that individuals believe, on Bayesian grounds, that conspiracies are more likely to be successful. As has been emphasized in the past (including by members of r/conspiracy), some conspiracy theories have proven to be true. As we noted above, for example, there is a relationship between conspiracy endorsement about medical experimentation among the African American community and awareness of actual abuses and cover-ups around the same issue. The conspiratorial mindset need not be read as wholly irrational: it may instead reflect awareness of actual past abuses of power. This is consistent with the “conspiratorial mindset” markers noted in the broad language analysis.

Notable over-representation by both user-count and post-count also occurred in the theme communities we labeled “Drugs and Bitcoin” and “Toxic Reddit”. The former includes a heterogeneous set of topics (including UFO and paranormal speculation) but can be characterized by a willingness to engage with socially “fringe” ideas of many kinds. The “Toxic Reddit” theme community also represents fringe engagement, but instead on the edges of acceptable taste. The most popular subreddits appear comparatively innocuous, but include r/KotakuInAction, which is a known hotbed of sexism and racism. Further, the subreddits in which r/conspiracy posters are also most over-represented include several that have since been banned for questionable content, such as r/WhiteRights and r/fatpeoplehate.

The inclusion of these subreddits suggests that the “conspiratorial mindset” tag may be in need of further refinement. On the one hand, it skirts tautology if read literally: claiming that people find a particular conspiracy attractive because they find conspiracy theories generally attractive carries relatively little explanatory power. On the other hand, the label may be overly restrictive. The more general affective consideration may be that conspiracy theories are outside of the mainstream of ordinary thinking, and that some people are attracted to a range of non-mainstream beliefs. That would assimilate conspiracy endorsement to a broader range of endorsements, which may in turn suggest novel lines of research.

Some of the discrepancies between our results and previous experimental studies may be due to differences in the population under study. In our analyses, we observed conspiracy engagement—users who were actively posting comments on stories in the r/conspiracy subreddit. Most experimental studies focus on willingness to *endorse* conspiracy theories, which appears to be more prevalent [11]. General powerlessness may make acceptance of conspiracy theories more attractive—but it requires a conspiratorial mindset to engage with and spread conspiracy theories in a social context. Taxonomizing individuals by the contents of their belief (i.e. by discussing “conspiracy theorists”) may thus be too coarse a cut for scientific purposes, and more fine-grained categorizations may be needed to capture the full dynamics of conspiracy endorsement. Our results suggest that people who are willing to discuss conspiracy theories in a social context are different from, or a special subset of the relatively broad populations who would endorse conspiracy theories when asked in isolation.

### Interactions

In the first two analyses, we identified the personal traits and social factors associated with future engagement with conspiracy beliefs. But these analyses are unable to shed light on whether, for example, r/conspiracy users appear angrier because they happen to be posting in subreddits which host particularly vigorous debates, or whether they exhibit anger in their posts even in the context of the social environments they inhabit.

A primary goal of the study was to disentangle self-selection effects from other cohort effects. Users from the r/conspiracy group differed from their matched cohort both in where they post and in the language they use in their posts. We interpreted the observed interaction between language and social factors as showing that this difference is primarily due to self-selection, rather than to the effect of either invariant traits or situational evocation.

Several patterns of interaction are theoretically possible. A complete lack of significant differences, especially in high-risk theme communities, would suggest that people self-select: that language use by r/conspiracy subjects is different because they tend to post in communities where that language finds a welcoming home. Conversely, consistent differences in the same linguistic factors across themes that vary in their association with eventual r/conspiracy posting (that is, in their user-count or post-count ratios) would suggest the importance of traits regardless of social communities. Differences in certain theme communities across factors would suggest that certain theme communities selectively enhance traits, possibly as part of a “radicalization” phenomenon. Finally, more complex patterns would suggest a more complicated causal story incorporating multiple processes.

The evidence for self-selection is twofold. First, across nearly all theme communities, there were relatively few significant differences in language use, and even fewer that met the pre-specified criterion we used in the first language use analysis. The lack of effect was most striking in the highest-risk theme communities like “Politics”. For example, r/conspiracy users discussed terrorism more than the control group, were more likely to post in political subreddits than the control group (and more often), but *within* the political subreddits their focus on terrorism is unremarkable. If the differences between the groups were due solely to extreme individual traits, we would expect to see language use differences persist when analyzed within the theme community.

Second, the exception to this general pattern is what we have dubbed the “Basic Reddit” theme. These are subreddits in which nearly everyone posts (i.e. the user-count ratio is close to 1.0). They are among the most popular subreddits (such as r/AskReddit, r/funny, and r/pics), and are generally innocuous in nature. Within this group, the language differences observed in the first analysis remained significant and strong in the third analysis. This suggests that the differences in the first analysis cannot be explained by situational factors because the differences between r/conspiracy users and the matched controls are still apparent within the subreddits that are most general.

The picture that best fits these observations is situational self-selection. In situational self-selection, individuals with a conspiratorial mindset select and post in subreddits where they appear relatively unremarkable. Further, this appears to be a process closer to the “choice mechanism” of Emmons et al. [56]: individuals appear to post just as stridently in subs where this would not necessarily be reinforced.

Of course, to say that the language within the politically themed subreddits is generally aligned with the social setting does not rule out the possibility that what is asserted is more conspiratorial in nature. Consider the following (deliberately obfuscated) comments from around the same time in politics-themed subreddits from an r/conspiracy user and their matched control:

r/conspiracy user: “Do you really deny that a politician might make decisions, after winning the race, that would help people who funded their campaign (or even to hurt people funded their opponents?) I’m not saying that only rich people win elections, I’m saying that money can corrupt political decision-making.”

matched control user: “The Tea Party movement took off when Glenn Beck began endorsing them. I am sure that MSNBC would cover a thoughtful left-wing counter-movement. What we would really need is enough push to make the movement credible, and then have some attractive faces in the media to promote it.”

Both quotes are concerned with questions of power, influence, and public perception. But the former is intuitively more suggestive of a conspiratorial mindset than the latter.

Finally, we note that the social environment analysis was relatively coarse-grained. Within each theme, there are certain subreddits where r/conspiracy posters are substantially over-represented when measured by user-count ratio. For example, the “Geek culture” theme includes subreddits such as r/collapse (devoted to discussion of “Resource depletion and ecological breakdown leading to the end of civilization”), r/WikiLeaks, and r/Anarchism. There were 8 times as many r/conspiracy group users posting in these subreddits as there were users from the matched control group. While the topics of the subreddits are aligned with traditional geek culture, they are more amenable to discussions related to conspiracy theories. The “Basic Reddit” theme community included subreddits such as r/Libertarian and r/MensRights, where there were 5 times as many r/conspiracy users as matched controls. The overall grouping still makes sense (as these are popular subreddits), but topics of discussion were also more likely to align with known conspiracy theories.

This raises the intriguing possibility of more fine-grained “gradients” within theme communities. Even when agents’ affiliations are driven entirely by self-selection, they face a discovery problem: it is not always obvious, especially in a crowded field, which groups will be most welcoming. To continue Funder’s [54] analogy: even if I like seedy bars, I might have trouble finding appropriately violent ones when I move to a new city. A good solution is word of mouth: I seek out the roughest bar I can find, and I find what the patrons already there say about other bars in the city. If there’s one that sounds more exciting, I try it. By iterating this process, I can eventually find action sufficient to my tastes.

**Fig 2:**
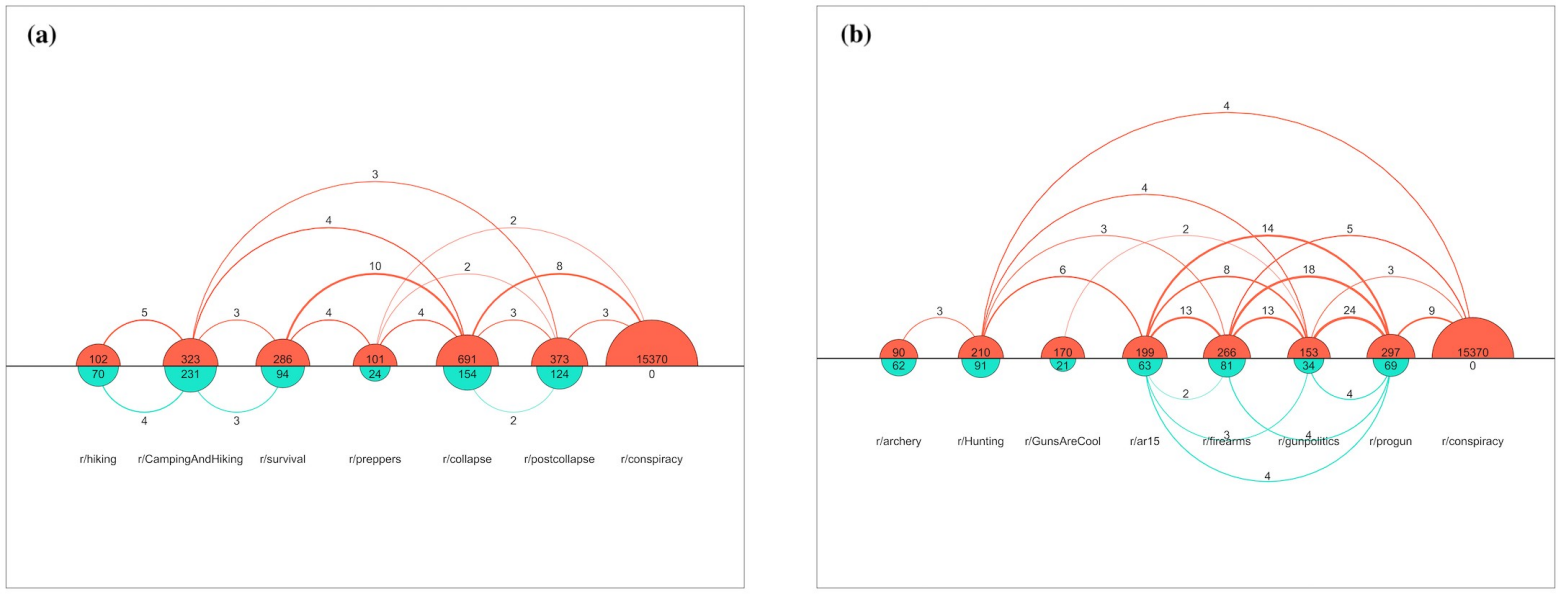
Chronological posting patterns. Examples of chronological posting patterns among r/conspiracy (orange, above) and control group (cyan, below) users for selected subreddits in the “Guns & Cars” theme community. Numbers within semi-circles are the total number of users posting in the subreddit; numbers above and below arcs are the number of users with contiguous chronological posts in two subreddits (from left to right).

Alfano, Carter, and Cheong [104] have dubbed this process “self-radicalization”. We suspect that a similar self-radicalization process may be at work in online forums. There is considerable traffic between, and discussion about, different subreddits. This word of mouth should aid the discovery process of new subreddits. Consider Fig 2, which shows chronological pathways of both r/conspiracy users and control group users through selected subreddits in the “Guns and Cars” theme community. Although the numbers are small, movement through increasingly risky subreddits towards r/conspiracy occurs more often in the r/conspiracy group compared to the control group.

### Limitations and future directions

Our study was subject to several methodological limitations. The dataset on which this study was based may have gaps in the availability of some of the comments from users, indicating that there is per-user risk of approximately 4% that one or more comments may be missing (the rate of missing comments is highest in 2009) [105]. However, since the study design relies on identifying differences between groups of users, missing data of this structure and magnitude would be unlikely to affect the results.

The dataset tracks Reddit *users* rather than individuals, and individuals can have multiple user accounts. To minimize this limitation, we constrained our set of participants to include only those users who posted with a minimum frequency over an extended period. Similarly, we only used information about users who were active participants in subreddits and could not determine whether users were reading forums without commenting (“lurking”). We think it is reasonable to take commenting to indicate active participation. However, we cannot rule out the possibility that some of our user accounts represent “alts”: that is, accounts made by individuals specifically to hide their less socially acceptable activities from searches. The presence of alts is a plausible explanation for the pattern observed in the “Pornography” theme community, which has a relatively high user-count ratio and a relatively low post-count ratio. However, we think the presence of alts would not fundamentally change our conclusions. Indeed, the maintenance of multiple social identities online might serve to aid self-selection of social groups, by reducing the need to mediate conflicts [106,107]. Large-scale online work may thus represent a valuable tool for looking at the negotiation of social identities.

Differences in language are useful but noisy proxies for psychological states. We note that other linguistic analyses have been used to study straightforwardly psychological phenomena. There are a range of other studies that use language markers from social media users to predict behavior changes relative to mental health conditions [108–110]. We think that the same logic readily extends to other, non-pathological psychological states. The robustness of our findings suggests that the results are a reasonable signal of differences in psychology.

Lexical analyses using a bag-of-words approach omit important context and can overlook subtle differences in *how* topics are discussed. The emotion-based Empath categories serve as something of a proxy for sentiment analysis, but proper sentiment analysis might give further distinguishing information. More powerful unsupervised methods such as topic modelling can also pick up differences in rhetorical and narrative style which differentiate different attitudes. Previous work on r/conspiracy suggests that skeptics could be differentiated by conspiracy endorsers by such means [62]. Developing more principled means of manual analyses of identified posters and comments might similarly aid interpretability.

Reddit is a global phenomenon with around 330 million monthly active users. As such, the sample is likely more diverse than many smaller experimental studies. The demographics of Reddit are skewed, however. Around 63% of Reddit users self-identify as male; 80% are between 18 and 35 years old; 82% are white; and 59% are single (See S1 Appendix). As such, while our findings are a reliable characterization of the population of Reddit users, we may not have reliably characterized engagement with conspiracy theories among minority populations [3]. However, further diversity would likely support rather than undermine our findings. In addition, Reddit is known to have played a role in spreading and amplifying misinformation from other parts of the web [80], suggesting that the importance of studying Reddit goes beyond the community itself.

We note again that because we studied Reddit users, we examined only people who have engaged with conspiracy theories in a social space, rather than the broader set of people who endorse or accept conspiracy theories. Both populations are important. We have suggested that some of the divergence between our findings and experimental results might reflect differences between these two groups. Experimental studies may be able to incorporate some of these insights by focusing on willingness to disseminate and discuss, rather than merely endorse, conspiratorial theories.

## Conclusion

Large-scale data analyses of online forums can shed light on how and why people engage with conspiracy theories. Results from analyses of what Reddit users post and where show that there are consistent language use differences between users who will eventually become engaged in a conspiracy theory forum compared to similar users who do not. The results also suggest that many of these differences in language are related to users actively selecting to engage with social groups whose interests and motives tend to fit with an incipient conspiratorial mindset. This does not rule out the possibility that further engagement with those groups ultimately helps to enhance conspiratorial leanings, but this would suggest amplification of existing biases rather than a *de novo* radicalization process. Further research would benefit from better understanding of the differences between people who endorse or accept conspiracy theories relative to those who engage with conspiracy theories in social spaces, as well as a deeper understanding of the confluence of personal traits and social circumstance that precedes engagement with conspiracy theories.

## Supporting information

S1 Appendix Appendix: Dataset availability and supporting details. (PDF)

S1 Fig The complete co-posting network. Node color represents degree of over-representation (blue = less, red = more). Node size represents absolute numbers of posters in the subreddit from the matched samples. Subreddits are organized via heuristic so that subreddits are closer to other subreddits with whom they are connected by larger overlap of users.
(TIFF)
