# Supplementary material for: Pathways to conspiracy: The social and linguistic precursors of involvement in Reddit’s conspiracy theory forum
Source: PLoS One. 2019 Nov 18;14(11):e0225098. doi: 10.1371/journal.pone.0225098 (PMC6860422; doi:10.1371/journal.pone.0225098)
Supplement: S1 Appendix — (PDF) [file pone.0225098.s001.pdf]

S1 Appendix: Dataset availability and supporting details

Data Availability

The reddit comment dataset was collected by Reddit user 'Stuck In The Matrix' using the official Reddit API. The post describing the dataset and its collection is available (as of 4 Nov 2019) at:
https://www.reddit.com/r/datasets/comments/3bxlg7/i_have_every_publicly_available_reddit_comment/

The dataset has been widely used for research purposes, and is available via academic torrents [1,2] at:
http://academictorrents.com/details/7690f71ea949b868080401c749e878f98de34d3d/tech

Please note that other versions of the dataset (such as those hosted by pushshift.io) may differ from the archived version used in our paper. Our ethics clearance does not permit hosting more specific intermediate versions of the dataset which might be able to identify particular users. Should academictorrents become unavailable in the future, please contact the authors and we will make reasonable arrangements to transmit the dataset.

[1] Henry Z. Lo. and Cohen, Joseph Paul "Academic Torrents: Scalable Data Distribution." Neural Information Processing Systems Challenges in Machine Learning (CiML) Workshop, 2016, http://arxiv.org/abs/1603.04395.

[2] Cohen, Joseph Paul, and Henry Z. Lo. "Academic Torrents: A Community-Maintained Distributed Repository." Annual Conference of the Extreme Science and Engineering Discovery Environment, 2014, http://doi.org/10.1145/2616498.2616528.

Reddit Demographics:

Demographic information was taken from the 2016 Reddit Demographics survey (the most recent available at the time of writing). A post describing this information can be found at: https://www.reddit.com/r/dataisbeautiful/comments/5700sj/octhe_results_of_the_reddit_demographics_survey/

The following demographic information is a description of the graphics associated with the above post, should they become unavailable in the future:

Age:

- 18-25 56.7%
- 26-35 22.7%
- <17 14.4%
- Other 6.2%

Gender:

- Male: 63.1%
- Female: 33.3%
- Other: 3.6%

Ethnicity:

- White: 81.8%
- Other: 18.2%

Bot Removal:

The list of known bots was downloaded from:

https://www.reddit.com/r/botwatch/comments/1xojwh/list_of_320_reddit_bots/

And combined with a list of users whose forum diversity (described in the text) was 15 Standard Deviations above the mean. The list of 466 usernames culled as bots is as follows:

A858DE45F56D9BC9
AAbot
ADHDbot
albumbot
allinonebot
AltCodeBot
ALTcointip
annoying_yes_bot
Antiracism_Bot
ApiContraption
A_random_gif
asmrspambot
AssHatBot
astro-bot
AtheismModBot
auto-doge
AutoInsult
automoderator
autourbanbot
autowikibot
AVR_Modbot
bad_ball_ban_bot
BadLinguisticsBot
BanishedBot
ban_pruner
baseball_gif_bot
beecointipbot
BeetusBot
BELITipBot
BensonTheBot
Bible_Verses_Bot
bitcoinpartybot
bitcointip
bitofnewsbot
BlackjackBot
BlockchainBot
bocketybot
__bot__
Brigade_Bot
bRMT_Bot
Bronze-Bot
c5bot
CAH_BLACK_BOT
Cakeday-Bot
callfloodbot
callibot
CalvinBot
canada_goose_tip_bot
CaptionBot
CarterDugSubLinkBot
CasualMetricBot
changetip
CHART_BOT
cheesecointipbot
Chemistry_Bot
ChristianityBot
chromabot
classybot
CLOSING_PARENTHESIS
Codebreakerbreaker
coinflipbot
coinyetipper
colorcodebot
Comment_Codebreaker
comment_copier_bot
compilebot
CompileBot
ComplimentingBot
conspirobot
CPTModBot
cRedditBot
creepiersmilebot
CreepierSmileBot
CreepySmileBot
cris9696
cruise_bot
CuteBot6969
d3posterbot
DDBotIndia
ddlbot
define_bot
DefinitelyBot
_Definition_Bot_
DeltaBot
demobilizer
dgctipbot
Dickish_Bot_Bot
Dictionary__Bot
DidSomeoneSayBoobs
digitipbot
disapprovalbot
DNotesTip
DogeLotteryModBot
dogetipbot
DogeTipStatsBot
DogeWordCloudBot
DotaCastingBot
Downtotes_Plz
DownvotesMcGoats
DRKTipBot
DropBox_Bot
earthtipbot
edmprobot
elMatadero_bot
elwh392
EmmaBot
Epic_Face_Bot
EscapistVideoBot
ExmoBot
expired_link_bot
ExplanationBot
fact_check_bot
faketipbot
FallacyBot
fa_mirror
FedoraTipAutoBot
fedora_tip_bot
Fedora-Tip-Bot
fedoratips
FelineFacts
Fixes_GrammerNazi_
flappytip
flips_title
foreigneducationbot
FriendlyCamelCaseBot
FriendSafariBot
FrontpageWatch
Frown_Bot
frytipbot
fsctipbot
FTFY_Cat
FTFY_Cat6
GabenCoinTipBot
gabenizer-bot
gabentipbot
GameDealsBot
Gatherer_bot
GATSBOT
GeekWhackBot
gfy_bot
gfycat-bot-sucksdick
GiantBombBot
GifAsHTML5
gifster_bot
givesafuckbot
gives_you_boobies
gocougs_bot
godwin_finder
golferbot
GoneWildResearcher
GooglePlusBot
GotCrypto
gracefulcharitybot
gracefulclaritybot
GrammerNazi_
GreasyBacon
gregbot
groompbot
Grumbler_bot
gunners_gif_bot
GunnersGifsBot
GunnitBot
GWHistoryBot
haiku_robot
Handy_Related_Sub
havoc_bot
HCE_Replacement_Bot
hearing_aid_bot
hearing-aid_bot
hearingaid_bot
HighResImageFinder
hit_bot
hockey_gif_bot
HockeyGT_Bot
HowIsThisBestOf_Bot
howstat
HScard_display_bot
hwsbot
IAgreeBot
I_BITCOIN_CATS
ICouldntCareLessBot
imgurHostBot
imgur_rehosting
imgurtranscriber
imirror_bot
Insane_Photo_Bot
I_Say_No_
IsItDownBot
IS_IT_SOLVED
isitupbot
jerkbot-3hunna
JiffyBot

JotBot
JumpToBot
KarmaConspiracy_Bot
Kevin_Garnett_Bot
keysteal_bot
kittehcointipbot
KSPortBot
last_cakeday_bot
LazyLinkerBot
Link_Correction_Bot
Link_Demobilizer
LinkedCommentBot
linkfixerbot1
linkfixerbot2
linkfixerbot3
LinkFixerBotSnr
Link_Rectifier_Bot
LocationBot
loser_detector_bot
luckoftheshibe
MAGNIFIER_BOT
Makes_Small_Text_Bot
makesTextSmall
malen-shutup-bot
malo_the_bot
matthewrobo
memedad-transcriber
meme_transcriber
Meta_Bot
MetatasticBot
MetricPleaseBot
Metric_System_Bot
misconception_fixer
mma_gif_bot
moderator-bot
MontrealBot
MovieGuide
MultiFunctionBot
MumeBot
NASCARThreadBot
Nazeem_Bot
nba_gif_bot
new_eden_news_bot
New_Small_Text_Bot
nfl_gif_bot
NFLVideoBot
nhl_gif_bot
Nidalee_Bot
NightMirrorMoon
NobodyDoesThis
NoSleepAutoMod
NoSobStoryBot2
not_alot_bot
notoverticalvideo
NotRedditEnough
NSLbot
nyantip
okc_rating_bot
pandatipbot
pandatips
PGN-Bot
PhoenixBot
PHOTO_OF_CAPTAIN_RON
PigLatinsYourComment
PJRP_Bot
PlaylisterBot
PlayStoreLinks_Bot
PleaseRespectTables
PloungeMafiaVoteBot
PokemonFlairBot
PoliteBot
PoliticBot
PonyTipBot
PornOverlord
Porygon-Bot
potdealer
PresidentObama___
ProselytizerBot
provides-id
PunknRollBot
QUICHE-BOT
qznc_bot
raddit-bot
randnumbot
Random-ComplimentBOT
RandomTriviaBot
Rangers_Bot
rarchives
Readdit_Bot
readsmalltextbot
Reads_Small_Text_Bot
RealtechPostBot
ReddCoinGoldBot
redditbots
redditreviewbot
reddtipbot
Relevant_News_Bot
relevantxkcd-bot
request_bot
RequirementsBot
RFootballBot
RfreebandzBOT
rhiever-bot
rightsbot
RiskyClickBot
Rita
rnfl_robot
roger_bot
r_PictureGame
rSGSpolice
rss_feed
rubycointipbot
rule_bot
rusetipbot
SakuraiBot
SakuraiBot_test
SatoshiTipBot
sentimentviewbot
serendipitybot
SERIAL_JOKE_KILLER
shadowbanbot
ShadowBannedBot
ShibeBot
ShillForMonsanto
Shiny-Bot
ShittyGandhiQuotes
ShittyImageBot
SketchNotSkit
slapbot
slickwom-bot
SmallTextReader
SMCTipBot
Smile_Bot
snapshot_bot
soccer_gif_bot
softwareswap_bot
Somalia_Bot
Some_Bot
sports_gif_bot
spursgifs_xposterbot
SRD_Notifier
SRScreenshot
SRS_History_Bot
StackBot
StarboundBot
stats-bot
steam_bot
StencilTemplateBOT
StreetFightMirrorBot
subtext-bot
SuchModBot
SurveyOfRedditBot
SWTOR_Helper_Bot
synonym_flash
tabledresser
techobot
tennis_gif_bot
test_bot0x00
Text_Reader_Bot
thankyoubot
TheSwedishBot
TipMoonBot
tipmoonbot1
tipmoonbot2
tips_bot
TitsOrGTFO_Bot
tittietipbot
topcoin_tip
TOP_COMMENT_OF_YORE
topredditbot
totes_meta_bot
ttumblrbots
TweetPoster
Twitch2YouTube
Unhandy_Related_Sub
unitconvert
UnobtaniumTipBot
UrbanDicBot
UselessArithmeticBot
UselessConversionBot
valkyribot
versebot
VerseBot
vertcoinbot
vertcointipbot
VideoLinkBot
VideopokerBot
VsauceBot
WeAppreciateYou
Website_Mirror_Bot
WeeaBot
wheres_the_karma_bot
WhoWouldWinBot
Wiki_Bot
Wiki_FirstPara_bot
WikipediaCitationBot
Wink-Bot
wooshbot
WordCloudBot2
WritingPromptsBot
wtf_content_bot
WWE_Network_Bot
X_BOT
xkcd_bot
xkcdcomic_bot
xkcd_number_bot
xkcd_transcriber
yes_it_is_weird
yourebot
YT_Bot
