# Supplementary figures and images for: Pathways to conspiracy: The social and linguistic precursors of involvement in Reddit’s conspiracy theory forum
Source: PLoS One. 2019 Nov 18;14(11):e0225098. doi: 10.1371/journal.pone.0225098 (PMC6860422; doi:10.1371/journal.pone.0225098)

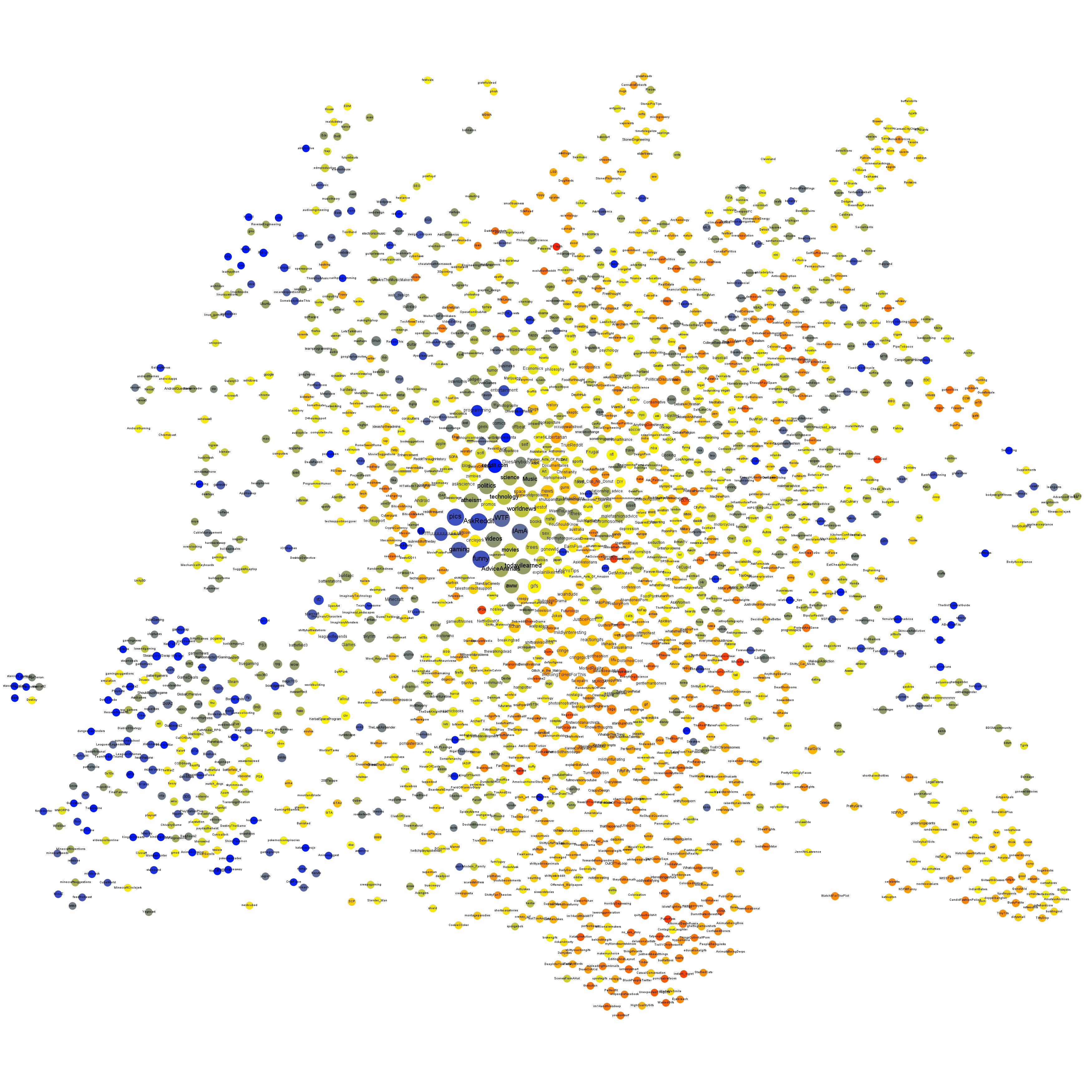

Supplement: S1 Fig — Node color represents degree of over-representation (blue = less, red = more). Node size represents absolute numbers of posters in the subreddit from the matched samples. Subreddits are organized via heuristic so that subreddits are closer to other subreddits with whom they are connected by larger overlap of users. (TIFF) [file pone.0225098.s002.tiff]
